# Orally administered *Odoribacter laneus* improves glucose control and inflammatory profile in obese mice by depleting circulating succinate

**DOI:** 10.1186/s40168-022-01306-y

**Published:** 2022-08-25

**Authors:** Isabel Huber-Ruano, Enrique Calvo, Jordi Mayneris-Perxachs, M-Mar Rodríguez-Peña, Victòria Ceperuelo-Mallafré, Lídia Cedó, Catalina Núñez-Roa, Joan Miro-Blanch, María Arnoriaga-Rodríguez, Aurélie Balvay, Claire Maudet, Pablo García-Roves, Oscar Yanes, Sylvie Rabot, Ghjuvan Micaelu Grimaud, Annachiara De Prisco, Angela Amoruso, José Manuel Fernández-Real, Joan Vendrell, Sonia Fernández-Veledo

**Affiliations:** 1 Hospital Universitari de Tarragona Joan XXIII, Institut d’Investigació Sanitària Pere Virgili, Tarragona, Spain; 2 CIBER de Diabetes y Enfermedades Metabólicas Asociadas (CIBERDEM)-Instituto de Salud Carlos III (ISCIII), 28029 Madrid, Spain; 3 Department of Diabetes, Endocrinology and Nutrition, Dr. Josep Trueta University Hospital, Girona, Spain; 4 Nutrition, Eumetabolism and Health Group, Girona Biomedical Research Institute (IdibGi), Girona, Spain; 5 Biomedical Research Networking Center for Physiopathology of Obesity and Nutrition (CIBEROBN), Madrid, Spain; 6 Rovira i Virgili University, 43003 Tarragona, Spain; 7 INRAE, AgroParisTech, Micalis Institute, Université Paris-Saclay, 78350 Jouy-en-Josas, France; 8 Department of Physiological Sciences, School of Medicine and Health Sciences, Nutrition, Metabolism and Gene therapy Group Diabetes and Metabolism Program, Institut d’Investigació Biomèdica de Bellvitge (IDIBELL), University of Barcelona, Barcelona, Spain; 9 Centro de Investigación Biomédica en Red Fisiopatología de la Obesidad y la Nutrición (CIBEROBN), Instituto de Salud Carlos III, 28029 Madrid, Spain; 10 Biomathematica, rue des Aloes, quartier Balestrino, Ajaccio, France; 11 Probiotical Research S.r.l., Enrico Mattei, 3, -28100 Novara, Italy

**Keywords:** Succinate, Obesity, Probiotics, Animal models, Inflammation, Glucose tolerance

## Abstract

**Background:**

Succinate is produced by both human cells and by gut bacteria and couples metabolism to inflammation as an extracellular signaling transducer. Circulating succinate is elevated in patients with obesity and type 2 diabetes and is linked to numerous complications, yet no studies have specifically addressed the contribution of gut microbiota to systemic succinate or explored the consequences of reducing intestinal succinate levels in this setting.

**Results:**

Using germ-free and microbiota-depleted mouse models, we show that the gut microbiota is a significant source of circulating succinate, which is elevated in obesity. We also show in vivo that therapeutic treatments with selected bacteria diminish the levels of circulating succinate in obese mice. Specifically, we demonstrate that *Odoribacter laneus* is a promising probiotic based on its ability to deplete succinate and improve glucose tolerance and the inflammatory profile in two independent models of obesity (*db/db* mice and diet-induced obese mice). Mechanistically, this is partly mediated by the succinate receptor 1. Supporting these preclinical findings, we demonstrate an inverse correlation between plasma and fecal levels of succinate in a cohort of patients with severe obesity. We also show that plasma succinate, which is associated with several components of metabolic syndrome including waist circumference, triglycerides, and uric acid, among others, is a primary determinant of insulin sensitivity evaluated by the euglycemic-hyperinsulinemic clamp.

**Conclusions:**

Overall, our work uncovers *O. laneus* as a promising next-generation probiotic to deplete succinate and improve glucose tolerance and obesity-related inflammation.

Video Abstract

**Supplementary Information:**

The online version contains supplementary material available at 10.1186/s40168-022-01306-y.

## Introduction

Obesity is an increasing global public health challenge and a major risk factor for developing pathologies such as type 2 diabetes, cardiovascular disease, and metabolic syndrome [1]. While a number of genetic and environmental factors contribute to the etiology of obesity, gut microbiota disturbances have recently emerged as an important determinant [2–4]. Indeed, microbial metabolites (e.g., short-chain fatty acids (SCFAs)) are now recognized as central messengers between the microbiota and the host, and their dysregulation is involved in the development of metabolic diseases [5].

The Krebs cycle intermediate succinate has the distinction of being produced by both the microbiota and the host [6]. In the context of microbiota metabolism, succinate has been traditionally considered as a cross-feeding intermediary metabolite in the microbial synthesis of the SCFA propionate [7]. While succinate has been long been perceived as an overall pro-inflammatory factor, even in the gut [8], it can also have beneficial effects on intestinal gluconeogenesis [9], and some authors have suggested the use of dietary succinate and bacterial succinate-producing species to improve glucose tolerance [10]. Indeed, a recent report revealed that succinate administration reduces chronic intestinal inflammation by modulating tuft cell expansion [11], which illustrates the complexity of host-microbiota metabolic interactions. Succinate is typically found in low concentrations in the gut lumen, likely related to its cross-feeding relationships, but some studies have suggested a link between the accumulation of luminal succinate and gut microbiota alterations associated with inflammatory bowel disease (IBD) or antibiotic treatment [6]. Along this line, we recently showed that obesity-related perturbations of gut microbiota are characterized by an increase in the abundance of bacterial succinate producers (*Prevotellaceae* and *Veillonellaceae*), concomitant with a reduction in succinate consumers (*Odoribacteraceae* and *Clostridaceae*) [12]. This specific microbiota signature was strongly associated with the higher circulating succinate observed in obesity. Nonetheless, no study has yet determined the origin of circulating succinate under steady-state or pathological conditions.

Beyond its role as a fuel metabolite, succinate is a signaling molecule responsible for many complex and often conflicting effects derived from its intracellular and extracellular actions, with the latter being particularly important in the regulation of the immune response. Accordingly, although succinate triggers divergent responses in a context- and tissue-dependent manner [13], it now appears fairly certain that the succinate/succinate receptor 1 (SUCNR1) axis fine-tunes immune cell biology to induce appropriate inflammatory responses [14]. Indeed, we recently demonstrated that SUCNR1 signaling in adipose tissue-resident macrophages is key for the resolution of acute inflammation––a physiological process that is inoperative in human obesity [15]. This may represent a novel mechanism underlying the chronic low-grade systemic inflammation evident in obesity. Notably, similar to what is seen with other hormones including insulin and leptin [16], obesity and type 2 diabetes are associated with higher circulating levels of succinate [12, 17] in a background of impaired SUCNR1 signaling (at least in terms of inflammation), which we have termed a “succinate-resistant” state. Whether this condition could be effectively counteracted by lowering circulating succinate levels as recently demonstrated for leptin [16] is unknown.

We sought to address the role of intestinal succinate in the context of obesity. To do this, we (i) explored the contribution of gut microbiota to systemic succinate, (ii) screened for non-pathogenic bacteria that could consume succinate in the human gut, (iii) tested whether oral treatment with such bacteria could effectively reduce circulating succinate and improve the metabolic and inflammatory status of obese mice, (iv) assessed circulating and fecal succinate in a cohort of patients with severe obesity, and (v) undertook metagenomic studies to identify specific associations between succinate and bacterial functions. Overall, our approach sets the stage for a new microbiota-mediated therapeutic strategy to reduce circulating succinate in patients with obesity and type 2 diabetes.

## Materials and methods

### In silico analysis

We first created a database of succinate-consuming bacteria based on the existing literature, data from the Virtual Metabolic Human Database (Noronha et al. 2019), and a BLAST search (sequence homology >80%, e-score < 0.00001) of the NCBI refseq database (release 200) for genes encoding propionyl-CoA:succinate CoA transferase (scpC, uniprot ID P52043), succinyl-CoA:CoA transferase (cat1, uniprot ID P38946), succinate dehydrogenase (PBF_RS12965, uniprot ID WP_035330248.1), or acetyl-coA hydrolase (HMPREF9453_RS07080, uniprot ID H1D192). From the list of bacteria retrieved, we included only those strains able to live in the human gut, without known pathogenic activity (based on literature and database searches including http://data.mypathogen.org/, https://www.patricbrc.org/, and http://www.mgc.ac.cn/VFs/main.htm), and for which genome assemblies were available for further analysis.

We then reconstructed the metabolic pathways of each bacterium from genome assemblies downloaded from NCBI (choosing the reference strain for each species) using the dedicated tool CARVEME, version 1.2.0 [18], and generated genome-scale metabolic models using the default parameters. We manually curated each genome-scale metabolic model according to the literature and dedicated databases (KEGG [19], UniProt [20], and JGI [21]) to ensure that they captured the available biochemical and physiological knowledge.

Similar to the studies of Monk et al. and Bosi et al. [22, 23], we then performed an in silico phenotypic screen to select species possessing succinate consumption phenotypes under different nutrient conditions, including some that prevail in the human gut. To do this, we performed flux balance analysis (FBA) simulations, a constraint-based genome-scale metabolic modeling approach [24, 25], for each genome-scale metabolic model obtained. FBA simulations were done using Matlab and the COBRA toolbox, version 3.0 [26]. We used the “optimizeCbModel” function with default parameters and changed the reaction bounds to −10 for limiting reactions, 0 for absent reactions, and <−20 for non-limiting reactions. For each species, we listed the nutrients that this species can use (based on the genome-scale metabolic models) and simulated the growth of each species in limiting conditions, all nutrients being limiting except one. We iteratively considered each nutrient as non-limiting (i.e., >20 mmol/gDW/h) while the others were limiting (i.e., <10 mmol/gDW/h). We determined the succinate consumption or production rates as well as the species growth rate in both aerobic and anaerobic conditions in each condition for each species. Finally, we performed simulations in human gut conditions using the western diet nutrient conditions [27].

### In vivo studies

All animal experiments were performed in accordance with ARRIVE guidelines, conforming to European Union Directive 86/609/EEC and recommendation 2007/526/EC regarding the protection of animals used for scientific purposes. For studies using germ-free (GF) mice, animal care procedures were approved by the ethics committee of the INRAE Research Center (approval reference: 17-14). Experiments using conventional mice were supervised and approved by the Parc Científic de Barcelona Ethical Committee for Animal Experimentation or the Universitat Rovira i Virgili Animal Welfare and Ethics Committee and later authorized by the Generalitat de Catalunya, Departament de Territori i Sostenibilitat, Direcció General de Polítiques Ambientals i Medi Natural with approval numbers 9201-P1 and 10283 and 11424, respectively.

### Germ-free mice

Four-week-old male and female GF and conventional C57BL/6J mice were used. GF mice were obtained from the breeding unit of Anaxem, the GF facility of the Micalis Institute (INRAE, Jouy-en-Josas, France; Anaxem license number: B78-33-6). Conventional mice were purchased from Charles River Laboratories (L'Arbresle, France). To maintain GF status, mice were housed in sterile isolators (Getinge, Les Ulis, France) and sterile conditions were monitored weekly by microscopic examination and screening cultures of freshly voided feces. Conventional mice were also maintained in isolators (non-sterile) to ensure the same environment between the two groups. Within the isolators, mice were kept in enriched home cages containing sterile bedding made of wood shavings, paper towels, and wooden sticks and had free access to autoclaved tap water and γ-irradiated (45 kGy) standard diet (R03; Scientific Animal Food and Engineering, Augy, France). The animal room was maintained at 20–24°C with a 12-h light/dark cycle (lights on at 7:30 am).

Twenty mice were used for each sex and bacterial status. All mice were housed individually for measurement of food consumption. Body weight and food were weighed once weekly from the age of 4 to 9 weeks. Mice were then anesthetized with isoflurane and killed by decapitation. Content and mucosa of the small intestine, cecum, and colon were collected, immediately frozen, and stored at −80°C for analyses.

### Dietary study

**Fig. 1:**
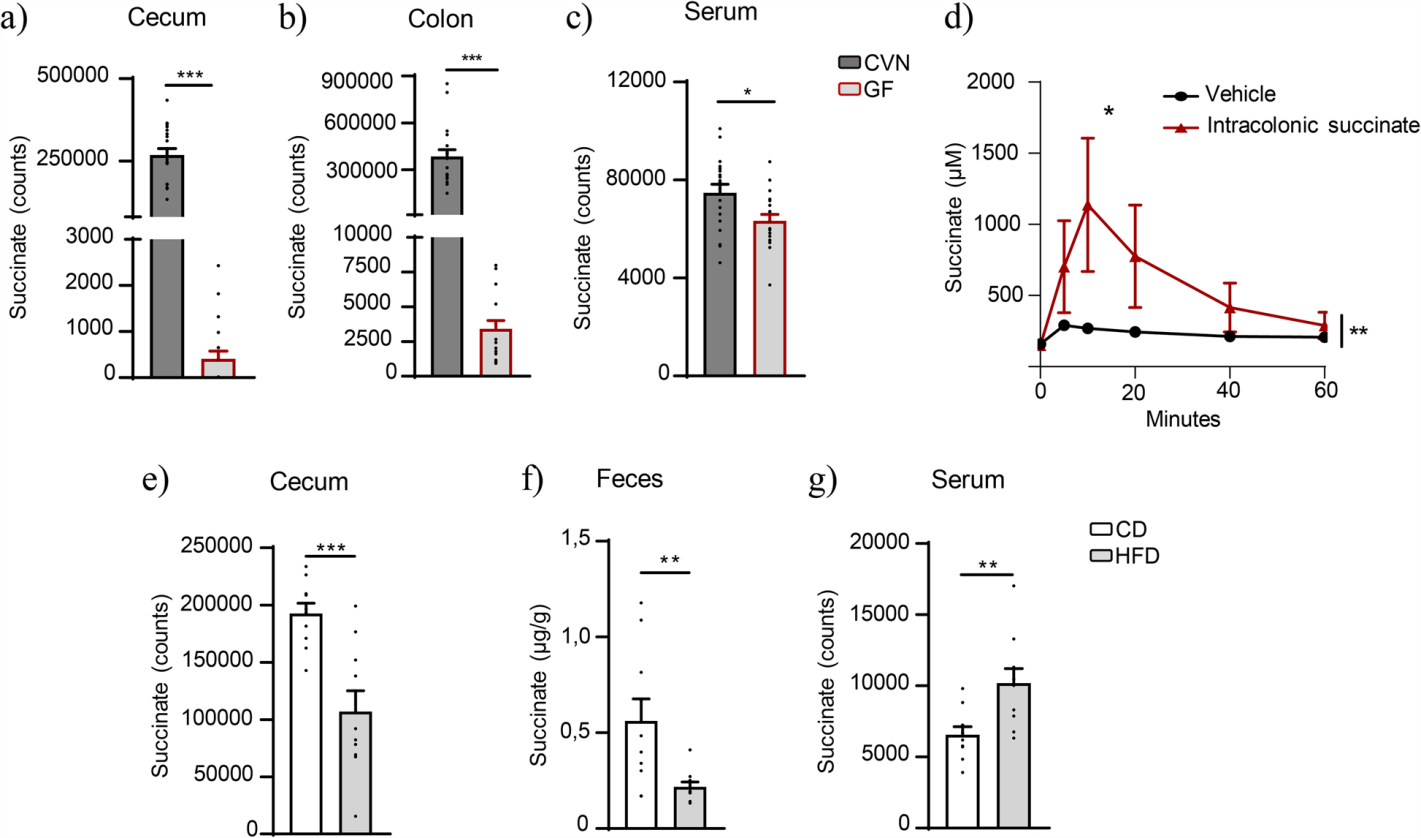
Gut microbiota and dietary regimen regulate intestinal and circulating succinate levels. Succinate levels in the cecum (**a**), colon (**b**), and serum (**c**) of conventional C57BL/6 (CVN) and germ-free (GF) mice (*n* = 16–19: males = 9–10; females = 7–9). Circulating succinate determination after intra-colon administration of 500 mg/kg of disodium succinate (or saline as vehicle) in C57BL/6 wild-type mice fed with chow diet (CD) (*n* = 4) (**d**). Succinate levels in the cecum (**e**), feces (**f**), and serum (**g**) of C57BL/6 mice fed high-fat diet (HFD) or CD (*n* = 10). Data are expressed as mean + s.e.m (**a**–**d**,**f**) or % over control (**e**). *p < 0.05; **p < 0.01; ***p < 0.001 (unpaired *t*-test and two-way ANOVA)

Two groups of ten 8-week-old male C57BL/6J mice (Charles River, Spain) were housed in standard conditions with a regular supply of water and food. For all dietary studies with the exception of the experiments described in Fig. 1e–g, one group was fed a high-fat diet (HFD, Research Diets D12451, 45% of kcal from fat) and the other group was fed a standard chow diet (CD, 8.4% kcal from fat [A04]; SAFE diets, Augy, France). Diets were maintained for 16 weeks. For the dietary study described in Fig. 1e–g, control animals were fed with 3.1% fat (SAFE diets, A04) and the other group with 60% fat (HFD, Research diets D12492) for 8 weeks. In all cases, cecal content, freshly deposited feces, and serum samples were obtained after sacrifice.

#### Intracolonic succinate administration

Succinate solution (50 μL at a dose of 500 mg/kg disodium succinate) or saline as vehicle was instilled into the colon of manually immobilized mice by inserting a fine round-tip cannula into the anus. Petroleum jelly was previously applied to the anal zone and the cannula. Blood was collected from the tail vein at the indicated time points.

#### Microbiota-depleted mice

Two groups of ten 8-week-old male C57BL/6J mice (Charles River, Spain) were housed under sterile conditions (as described above) in air and temperature-controlled cages with a regular supply of water and food. Animals were fed for 16 weeks with HFD. We then depleted the microbiota in one group using a described procedure [28]. Briefly, a cocktail of the following antibiotics was added to the drinking water for 3 weeks: 250 mg/L vancomycin, 500 mg/L metronidazole, 250 mg/L neomycin, 50 mg/L streptomycin, 170 mg/L gentamycin, 125 mg/L ciprofloxacin, 100 mg/L ceftazidime, and 1 g/L bacitracin. The cocktail was used at 25% of the initial concentration in the 3rd week. Mice were fed with HFD until sacrifice, and their body weight and food intake were measured twice weekly. Cecal content, freshly deposited feces and serum samples were obtained after sacrifice.

### Probiotic intervention

#### Experiment 1

Eight-week-old male *db/db* mice (Charles River, Spain) fed CD were distributed into seven experimental groups and treated as follows: vehicle (PBS+10% glycerol), *Odoribacter laneus* DSM 22474 (DSMZ, German Collection of Microorganisms and Cell Cultures), heat-inactivated (100°C for 30 min) *O. laneus* DSM 22474, *Clostridium butyricum* DSM 10702, *Dialister hominis* DSM 109768, *Dialister succinatiphilus* JCM 15077 (JCM, Japan Collection of Microorganisms), and *Phascolarctobacterium succinatutens* DSM 22533. Treatments were administered by oral gavage (1–5×10^8^ cfu) for 3 weeks on alternate days.

#### Experiment 2

Eight to 9-week-old C57BL/6 male mice (Charles River, Spain) were fed HFD (D12451) for 16 weeks and then randomly divided into a control (vehicle) group, a *O. laneus*-treated group, and a heat-inactivated *O. laneus*-treated group. Animals were subjected to the same experimental conditions as in experiment 1.

#### Experiment 3

Eight to 9-week-old male *Sucnr1* knock-out mice on a C57BL/6 background [29], and equivalent C57BL/6 male mice were fed 60% kcal HFD (Research diets D12492) for 8 weeks and treated with live *O. laneus* using the same conditions as described for experiment 1.

### Glucose and insulin tolerance tests

For the glucose tolerance test, mice were administered with an intraperitoneal load of glucose solution (2 g glucose/kg body weight) after a 16-h fast. Glucose levels were determined from blood samples taken from the tail vein at 0, 15, 30, 60, 90, and 120 min using a handheld glucometer (Accu-Chek glucose reader; Roche, Mannheim, Germany). Insulin levels in response to glucose were measured at 0 and 30 min post-injection using a commercial mouse insulin ultrasensitive enzyme-linked immunosorbent assay (BioVendor Research and Design products, Brno, Czech Republic). For intraperitoneal insulin tolerance analysis, mice were fasted for 3 h, after which they were injected with insulin (0.75 U/kg). Glucose was measured as described above.

### Succinate determination

Excluding the experiments using GF mice, serum samples from 16-h fasted mice and plasma/fecal samples from the human cohort (see later) were filtered (10 kDa) and succinate levels were determined using a fluorometric assay (EnzyChrom Succinate Assay Kit; BioAssay Systems, Hayward, CA).

For GF cecal and colon content and serum analysis, we used liquid chromatography with triple-quadruple mass spectrometry (LC-QqQ MS) to measure succinate. Succinate was extracted by mixing 300 μL or 400 μL of acetonitrile/methanol/water (4:4:2 vol) with 25 μL of serum or ~25 mg of intestinal content (colon and cecum), respectively, followed by vortexing. For the serum analysis, the mixture was incubated at −20°C for 1 h. For the intestinal content, the mixtures were frozen in liquid nitrogen for 10–20 s, then bath-sonicated for 20–30s, and vortexed for 20 s. These steps were repeated three times. Targeted analysis of succinate was performed using an ultra-high-performance LC system coupled to a 6490 QqQ mass spectrometer (Agilent Technologies, Palo Alto, CA) with iFunnel technology in negative electrospray ionization (ESI). Succinate was retained and separated by hydrophilic interactions using an InfinityLab Poroshell 120 HILIC-Z, 2.1 × 100 mm, 2.7 μm (PEEK lined) (Agilent Technologies) column. Mobile phase A was 50 mM ammonium acetate and 5 μM medronic acid in water and mobile phase B was acetonitrile. Five microliters of the extracts was injected into the LC-MS/MS system. Peak area was manually integrated using Mass Hunter Qualitative software (Agilent Technologies).

### Succinate consumption assay

In order to test the ability of anaerobic strains to metabolize succinate, bacteria were grown on modified yeast casitone fatty acids (YCFA) supplemented with 1% sodium succinate (Sigma, S9637). Growth medium was constituted as follows: trypticase peptone (10g/L), yeast extract (2.5 g/L), glucose (5 g/L), MgSO_4_ (45 mg/L), CaCl_2_ (90 mg/L), K_2_HPO_4_ (0.45 mg/L), KH_2_PO_4_ (0.45 mg/L), NaCl (0.9 g/L), NaHCO_3_ (4 g/L), L-cysteine HCl (1.0 mg), sodium succinate (10 g/L), acetic acid (1.90 mL/L), propionic acid (0.70 mL/L), and 1% vitamin solution (2 mg/L biotin, 2 mg/L folic acid, 10 mg/L pyridoxine-HCl, 5 mg/L thiamine HCl, 5 mg/L riboflavin, 5 mg/L nicotinic acid, 0.10 mg/L cobalamin, 5 mg/L p-aminobenzoic acid). Resazurin solution (500 ppm) was always used in the medium as an oxidation-reduction indicator. Cultivation broth was distributed in serum bottles and flushed extensively under an anaerobic gas mix (90% N_2_, 5% CO_2_, 5% H_2_) before sterilization at 121°C × 15 min. Subsequently, the pH was adjusted to 7.0 **±** 0.1 with 1N NaOH. All the strains were handled for growth experiment, incubated, and prepared for enumeration analysis in an anaerobic workflow (Bactron 300-2, Kentron Microbiology) in order to ensure anaerobic conditions. For inoculum standardization and cell load determination, the number of cells individuated by the flow cytometer as active fluorescent units (AFU)/mL was considered. After the first incubation in succinate YCFA for cell activation, bacterial cultures were sub-cultured twice in measure of 1 × 10^6^ AFU/mL in fresh medium. Cell load (AFU)/mL was constantly monitored, and growing experiments were terminated when all the strains reached in broth a cell density ranging from 100 to 300 MLN AFU/mL. Subsequently, freshly grown cultures were centrifuged at 7000 rpm × 8 min to recover the supernatant that was subsequently filter sterilized with 0.22-μn membrane filter. Cell-free supernatant was kept at −20 °C before being analyzed for succinate measurement.

### Short-chain fatty acid fecal determination

Samples (20 mg fecal matter) were placed in a centrifuge tube and mixed with 200 μL of internal standard (butyric-1,2-13C2 acid and propionic d6 acid at 100 mg/L and sodium acetate 13C2 at 500 mg/L) and 200 μL of PBS. Samples were vortexed for 2 min and centrifuged at 15,000 rpm/4°C for 5 min. Supernatants (50 μL) were acidified with 10 μL of 15% phosphoric acid, followed by the extraction of SCFAs using 1000 μL methyl tert-butyl ether. Samples were centrifuged and the upper layers (organic) were transferred to glass vials for gas chromatography (GC) analysis.

For GC-(EI)-MS/MS analysis, a volume of 1.5 μL of the sample was automatically injected into a split/splitless inlet in splitless mode, maintained at 250°C. Helium (99.999% purity) was used as a carrier gas, at a flow rate of 2.25 mL/min. The oven program was set at an initial temperature of 40°C, increased to 130°C at a rate of 12°C/min, then increased to 200°C at a rate of 30°C/min, then increased to 250°C at a rate of 100°C/min. Ionization was done by electronic impact (EI), with an electron energy of 70eV and a source temperature of 250°C. Mass spectra data were recorded after a solvent delay of 5 min. The mass spectrometer analyzer was operated in multiple reaction monitoring mode.

### 16S rRNA analysis from mouse samples

16S rRNA gene amplicons were obtained following the 16S rRNA gene sequencing Library Preparation Illumina protocol (Illumina Inc., San Diego, CA; Cod. 15044223 Rev. A). The gene-specific sequences used in this protocol target the 16S rRNA gene V3 and V4 regions. Illumina adapter overhang nucleotide sequences were added to the gene-specific sequences. The primers were selected as described [30]. The full-length primer sequences, using standard IUPAC nucleotide nomenclature, targeting this region are 16S rRNA V3-V4 Forward: TCGTCGGCAGCGTCAGATGTGTATAAGAGACAGCCTACGGGNGGCWGCAG; 16S rRNA V3-V4 Reverse: GTCTCGTGGGCTCGGAGATGTGTATAAGAGACAGACTACHVGGGTATCT AATCC.

We used microbial genomic DNA (5 ng/μL in 10 mM Tris, pH 8.5) to initiate the protocol. After 16S rRNA gene amplification, the multiplexing step was performed using the Nextera XT Index Kit (Illumina; FC-131-1096). PCR products (1 μL) were run on a Bioanalyzer DNA 1000 chip to verify the size; the expected size on a Bioanalyzer trace is ~550 bp. After size verification, the libraries were sequenced using a 2×300 bp paired-end run (MiSeq Reagent kit v3 [MS-102-3001]) on a MiSeq Sequencer according to the manufacturer’s instructions (Illumina).

Data were obtained using an *ad-hoc* pipeline written in RStatistics environment (R Core Team, 2012), making use of several Open Source libraries, and the sequence data were analyzed using the qiime2 pipeline [31]. Metataxonomy analysis was performed using qiime2 plugins. Denoising, paired-end joining, and chimera depletion were performed starting from paired-end data using the DADA2 pipeline [32]. Taxonomic affiliations were assigned using the Naive Bayesian classifier integrated in quiime2 plugins. The database used for this taxonomic assignation was the Silva138 [33]. Microbial counts were stored through phyloseq (version 1.36.0) and differential Abundance Analysis was performed using Analysis of Compositions of Microbiomes with Bias Correction (ANCOM-BC 1.2.2), which accounts for the compositional nature of the data and has shown to be a conservative, high-precision tool in multiple benchmarks [34]. Top 10 abundant taxa for relative abundance plots were selected with tidyMicro (1.4.7) and those taxa representing at least 0.2% of the total were plotted. For visualizations, PCA was conducted over CLR transformation with compositions (2.0-4) through FactoMineR (2.4).

### Gene expression analysis

Total RNA was extracted from tissues using the RNeasy Lipid Tissue Mini Kit (Qiagen Science, Hilden, Germany). RNA was converted to cDNA using the Reverse Transcription System (Applied Biosystems, Carlsbad, CA), and quantitative gene expression was analyzed by real-time PCR (RT-qPCR) on a 7900HT Fast Real-Time PCR System using the TaqMan Gene Expression Assay (Applied Biosystems). Details on the probes are provided in Supplementary Table S1: Additional File 17. *B2m* was used as the housekeeping gene and the relative expression was calculated using the comparative Ct method (2-ΔΔCt).

### Clinical study design

Twenty-five patients with severe obesity (body mass index [BMI] 44.69 ± 4.53) were included in the study (see Table 1). Participants were recruited at the Endocrinology Service at the University Hospital Dr. Josep Trueta (Girona, Spain). All subjects were of Caucasian origin and reported a stable body weight and received no antibiotics, probiotics, prebiotics, or any other medical treatment influencing intestinal microbiome in the 3 months prior to the study. All patients had fasted overnight before the collection of blood and stool. Plasma, fecal, and intestinal (jejunum) samples were collected in all subjects. The jejunum was obtained during bariatric surgery (gastric bypass). The following exclusion criteria were considered: (i) no systemic inflammatory disease other than obesity; (ii) free of any infections in the previous month before the study; and (iii) no liver diseases (specifically tumor disease and infections) or thyroid dysfunction, which would be specifically excluded by biochemical work-up. The protocol was revised, validated, and approved by the Ethics committee of the Hospital Dr Josep Trueta (Project number 2019.143, September 24, 2019). The purpose of the study was explained to participants and all signed a written informed consent before being enrolled in the study.

**Table 1:** Clinical and laboratory data of the human cohort

	All cohort *N* = 25
Sex (female/male), *n*	21/4
Age (years)	45.46±8.33
Weight (kg)	113.62±22.38
BMI (kg/m^2^)	44.69±4.53
Waist circumference (cm)	124.60±11.65
Hip circumference (cm)	136.16±12.20
Waist-to-hip ratio	0.92±0.10
Systolic blood pressure (mmHg)	140.44±19.39
Diastolic blood pressure (mmHg)	79.80±12.07
Glucose (mg/dL)	99.08±12.38
Insulin (pmol/L)	30.32±16.01
HbA1c (%)	5.46±0.35
*M*-value	4.64±2.62
Total cholesterol (mg/dL)	179.84±27.55
HDL cholesterol (mg/dL)	48.40±8.37
LDL cholesterol (mg/dL)	111.08±23.48
Triglycerides (mg/dL)	101.72±40.94
ALT (U/L)	28.17±17.17
AST (U/L)	23.56±13.97
GGT (U/L)	42.00±44.17
Uric acid (mg/dL)	5.50±1.34

Data are presented as proportion or mean±SD*. BMI* Body mass index, *HbA1c* Glycated hemoglobin, *HDL* High-density lipoprotein, *LDL* Low-density lipoprotein, *ALT* Alanine aminotransferase, *AST* Aspartate aminotransferase, *GGT* Gamma-glutamyl transferase

#### Laboratory determinations in the human cohort

Blood samples were drawn after a 12-h fast. Plasma was separated and immediately frozen at −80 °C. Glucose and lipid profiles were measured by standard enzymatic methods (CobasR 8000 c702, Roche Diagnostics, Basel, Switzerland). Plasma insulin was analyzed by immunoassay (Coat-A-Count Insulin; Diagnostic Products, Los Angeles, CA). Succinate was determined in plasma/feces filtrates as described above. Glycated hemoglobin (HbA1c) was determined by high-performance liquid chromatography (ADAMRA1c HA-8180V, ARKRAY, Inc., Kyoto, Japan). Insulin sensitivity (M-value) was assessed using the euglycemic-hyperinsulinemic clamp and was defined as the weight-adjusted glucose infusion rate during the last 30 min when a steady-state was achieved [35]. The main anthropometric and clinical variables are described in Table 1.

### Extraction of genomic DNA from human fecal samples and whole-genome shotgun sequencing

Total DNA was extracted from frozen human stool using the QIAamp DNA mini stool Kit (Qiagen). Quantification of DNA was performed with a Qubit 3.0 fluorometer (Thermo Fisher Scientific, Carlsbad, CA), and 1 ng of each sample (0.2 ng/μL) was used for shotgun library preparation for high-throughput sequencing, using the Nextera DNA Flex Library Prep kit (Illumina). Sequencing was carried out on a NextSeq 500 sequencing system (Illumina, action co-financed by the European Union through the Operational Program of the European Regional Development Fund [ERDF] of the Valencian Community 2014–2020) with 2×150-bp paired-end chemistry, at the Sequencing and Bioinformatic Service of the FISABIO (Valencia, Spain). The input fastq files were decompressed, filtered, and preprocessed using the prinseq-lite-0.20.4 program, and overlapping pairs were joined using FLASH-1.2.11. Fastq files were then converted into fasta files, and human host reads were removed by mapping the reads against the reference human genome (GRCh38.p11; Dec 2013) using bowtie2-2.3.4.3 with end-to-end and very sensitive options. Taxonomic annotation was implemented with Kaiju v1.6.2.

Functional analyses were performed by assembling the non-host reads into contigs using MEGAHIT v1.1.2 [36] and mapping those reads against the contigs with bowtie2. Reads that did not assemble were appended to the contigs. Next, Prodigal v2.6.342 [37] was used for predicting codifying regions. Functional annotation was carried out with HMMER [38] against the Kyoto Encyclopedia of Genes and Genomes (KEGG) database, version 2016 [19] to obtain the functional subcategory, route, and annotation of the genes. Filtering of the best annotations and assignment of the ORF annotation to every read was carried out using the statistical package R v3.1.0 (R Development Core Team, 2013), which was also used to count the aligned reads and to add the category and its coverage, and finally to build abundance matrices. Addition of lineage information was added, and counting of taxa and generation of an abundance matrix for all samples was performed using R.

### Statistical analysis

In vitro data are expressed as mean ± s.e.m. Differences between the two groups were determined using Student’s *t*-test or the Mann-Whitney *U* test (two-tailed, 95% confidence interval) after data normality checking. Statistical significance of body weight and glucose and insulin tolerance tests was tested using a two-way analysis of variance. A *p*-value < 0.05 was considered statistically significant. All statistical analyses were performed using GraphPad Prism 6.0 software (GraphPad Inc., San Diego, CA).

For clinical data, the relationship between plasma and fecal or plasma succinate and anthropometric and clinical variables was analyzed using Kendall’s rank correlation coefficients. Linear regression analyses were performed to identify which variables were independent factors of the M-value. All variables associated in the univariate analysis with the dependent variable were included in the model as potential independent variables. Regression analyses were performed with SPSS 27 software (IBM, Armonk, NY).

For metagenomic data, associations between glucose, insulin, homeostatic model assessment of insulin resistance (HOMA-IR), and M-value and different bacterial species were also assessed using Kendall’s tau correlation coefficients. To consider the compositional structure of the microbiome data and rule out possible spurious associations, raw counts were transformed using a centered log-ratio (clr) transformation, as implemented in the “ALDEx2” R package, before correlation analyses. It first uses a Dirichlet-multinomial model to infer abundance from read counts and then applies a clr transformation to each instance. We used 128 Dirichlet Monte Carlo instances in the aldex.clr function. Succinate levels were used as a continuous variable in the models for each taxon or KEGG orthologue. All variables associated in the univariate analysis with the dependent variable were included in their respective models as potential independent variables. Regression analyses were performed with SPSS 26 software (IBM, Armonk, NY).

## Results

### Gut microbiota is a relevant source of succinate in circulation, particularly in an obesity setting

We first assessed the contribution of gut microbiota to circulating succinate by measuring its levels in fecal matter from the cecum and colon, and in the serum, from fasted GF and conventional mice. Results showed that the absence of microbiota was linked to a markedly lower concentration of intestinal succinate (cecum and colon) (Fig. 1a, b). Moreover, circulating succinate was moderately but significantly lower in GF mice than in conventional mice (Fig. 1c), indicating that microbiota contributes, at least partly, to circulating succinate in fasting conditions. To further confirm the ability of succinate to cross the intestinal barrier, we intra-colon administered succinate (500 mg/kg) or saline (as vehicle) to conventional mice and we observed a peak in circulating succinate 10 min after administration (Fig. 1d).

Circulating succinate is elevated in obesity [12] and it is widely known that obesity is associated with a modification of the gut microbiome that alters intestinal permeability, favoring the translocation of metabolites to circulation [39]. In line with this notion, we found that succinate levels were significantly lower in the cecum content (Fig. 1e) and the feces of mice on HFD (mimicking diet-induced obesity [DIO]) than on CD (0.22 ± 0.02 μg/g in HFD vs. 0.56 ± 0.11 μg/g in CD samples; *p* = 0.009; Fig. 1f) and this was accompanied by significantly higher levels of circulating succinate (Fig. 1g).

**Fig. 2:**
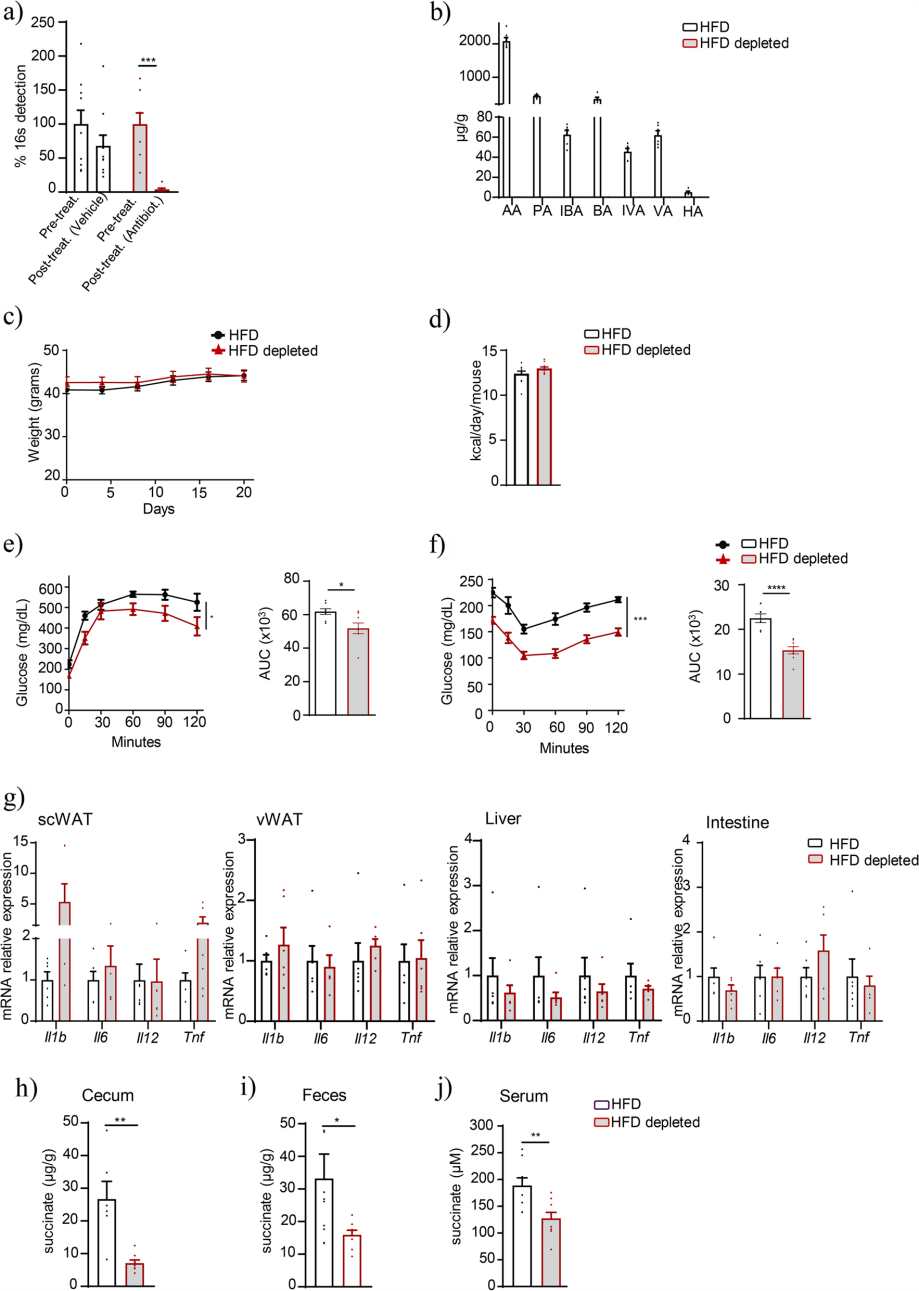
Microbiota depletion improves glucose metabolism and reduces succinate levels in C57BL/6 diet-induced obese mice. Percentage of 16S rRNA gene detection in cecum before and after antibiotic or vehicle administration (**a**). Cecal short-chain fatty acid analysis: acetic acid (AA), propionic acid (PA), butyric acid (BA), indolebutyric acid (IBA), isovaleric acid (IVA), valeric acid (VA), and hexanoic acid (HA) (**b**) (*n* = 6–8). Body weight evolution (**c**). Food consumption (**d**). Glucose (**e**) and insulin (**f**) tolerance tests (*n* = 8–10). mRNA expression levels of inflammatory genes in the scWAT, vWAT, liver, and intestine (**g**) (*n* = 6). Succinate levels in the cecum (**h**), feces (**i**), and serum (**j**) (*n* = 6–8). Data are presented as mean + s.e.m. *p < 0.05; **p < 0.01; ***p < 0.001 (unpaired *t*-test and two-way ANOVA)

We next sought to determine the contribution of gut microbiota to circulating succinate in the context of obesity. Because the GF model is resistant to DIO [40], we used conventional mice on HFD as a model of DIO and subsequently depleted the microbiota with a cocktail of broad-spectrum antibiotics for 3 weeks. Microbiota depletion was confirmed by 16S rRNA sequencing (Fig. 2a) and by measuring the levels of fecal SCFAs, which were almost undetectable in the microbiota-depleted group relative to the control (vehicle) group (Fig. 2b). Mice in both groups showed similar body weight evolution and food consumption (Fig. 2c, d) and, as expected [41], microbiota depletion induced a significant improvement in glucose tolerance (Fig. 2e) and insulin sensitivity (Fig. 2f). Because microbiome perturbations have been previously linked to exaggerated inflammatory responses in some organs [42, 43], we measured the expression of several key inflammatory genes in different tissues. Microbiota depletion induced a diverse response in different organs, with a non-significant increase in the expression of some pro-inflammatory cytokines (*Il1b* and *Tnf*) in subcutaneous white adipose tissue (scWAT) and an overall decrease in the liver (Fig. 2g). No changes were detected in visceral WAT (vWAT) or intestine, suggesting that the inflammatory effects of microbiota depletion are tissue-specific. Finally, we assessed succinate levels both in feces and serum, finding that succinate was markedly reduced in the samples from the cecum (26.67 ± 5.45 μg/g in control mice on HFD vs. 7.12 ± 0.94 μg/g in microbiota-depleted mice on HFD; *p* = 0.0015; Fig. 2h) and feces (33.17 ± 7.52 μg/g in control mice on HFD vs. 15.92 ± 1.45 μg/g in microbiota-depleted mice on HFD; *p* = 0.03; Fig. 2i) and that circulating succinate levels were also reduced by 33% after microbiota depletion (188.9 ± 14.16 μM in HFD vs. 127.4 ± 11.15 μM in microbiota-depleted HFD samples; *p* = 0.004; Fig. 2j).

Overall, these data demonstrate that microbiota depletion improves the metabolic profile of obese mice concomitant with a reduction in succinate levels. Notably, the data point to a commensal microbiota-derived origin of succinate in an obesity setting.

### Probiotic intervention with *Odoribacter laneus* reduces circulating succinate and ameliorates obesity-related inflammation

We tested the possibility of reversing succinate resistance and restoring its anti-inflammatory function using a therapeutic strategy based on probiotic interventions with succinate-consuming bacterial strains to deplete circulating succinate. As there is no clear classification of bacteria based on succinate metabolism, we created a database of succinate-consuming bacteria using the existing literature, data from the Virtual Metabolic Human Database [44], and from a BLAST search of the NCBI refseq database, release 200, for genes involved in succinate consumption (see the “Materials and methods” section), which identified 89 potential succinate-consuming bacterial strains. From this preliminary list, we discarded bacteria that had known pathogenic activity reported in different databases or in the scientific literature, which generated a final list of 22 candidates (Supplementary Table S2: Additional File 17). We then performed FBA [24, 25] of the 22 bacteria in non-limiting conditions, with each metabolite removed from the medium individually in both aerobic and anaerobic conditions. From this analysis, *Odoribacter laneus* and *Selenomonas felix* showed a certain degree of succinate consumption ability in several different limiting conditions and only *Dialister succinatiphilus* showed a clear succinate-consuming phenotype in almost all limiting conditions. No other bacteria showed a succinate-consuming phenotype in this analysis. Finally, we performed simulations in human gut conditions using the western diet nutrient conditions to select species possessing the phenotypic trait of succinate consumption and we confirmed that *D. succinatiphilus*, *O. laneus*, and *S. felix* consumed succinate in gut conditions in silico.

**Fig. 3:**
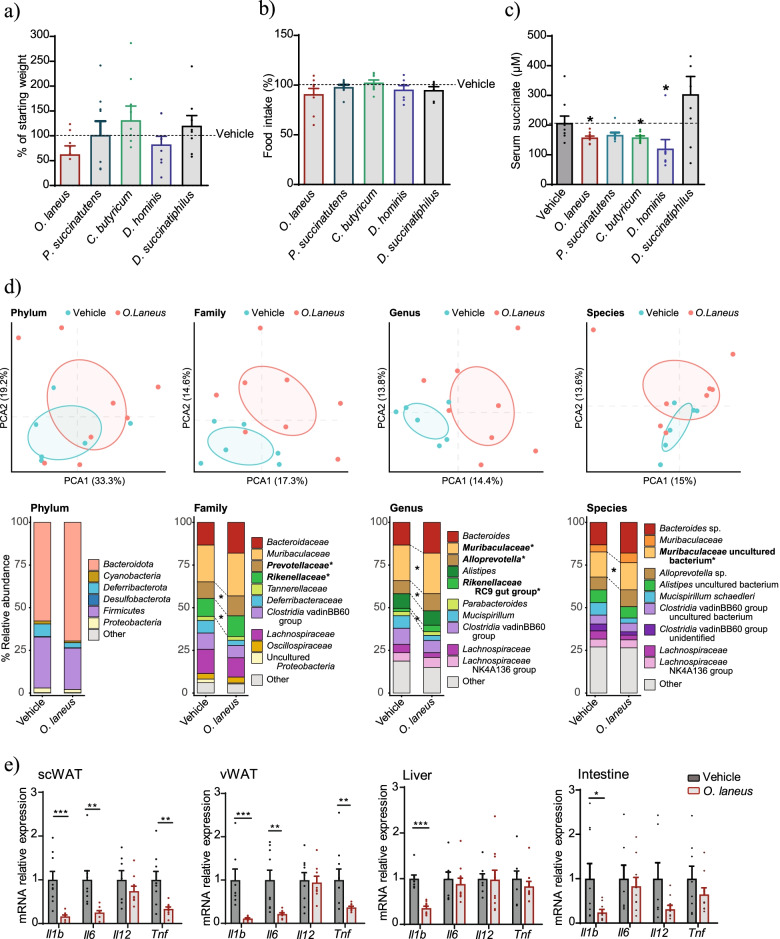
Probiotic intervention with *Odoribacter laneus* depletes serum succinate and moderates inflammation in *db/db* mice. Effect of different probiotic interventions on body weight (**a**), food intake (**b**), and fasted serum succinate levels (**c**) (*n* = 8–10). Principal component analysis and average phylum, family, genera, and species abundance of the 10 more abundant taxa in the cecum of *db/db* mice treated with vehicle or *O. laneus* (**d**) (*n* = 15). mRNA expression levels of inflammatory genes in the scWAT, vWAT, liver, and intestine (**e**) (*n* = 7–10). Data are presented as mean + s.e.m. *p < 0.05; **p < 0.01; ***p < 0.001 (unpaired *t*-test)

At this point, we discarded *S. felix* from further study, as the strain was not commercially available. Also, while constraint-based methods such as FBA have considerable predictive capability [26, 45], our approach only gave very limited information, and we decided not to restrict the in vivo screening to the data obtained from the computational approach and to include other representative well-described bacteria for which succinate-consumption ability has been demonstrated in vitro, such as *Phascolarctobacterium succinatutens* [46] and *D. hominis* (not identified in the FBA analysis) [47], or *in vivo* such as *Clostridium butyricum* [48]. All strains were tested by oral gavage in *db/db* obese mice on alternate days for 3 weeks. No significant differences in body weight or food consumption were observed for any of the strains tested (Fig. 3a, b), and only *O. laneus* administration showed a trend to lower body weight evolution (Fig. 3a). With the exception of *P. succinatutens and D. succinatiphilus*, all were able to lower circulating succinate levels in *db/db* mice (158.2 ± 5.11 μM for *O. laneus* (*p* = 0.02); 158.6± 5.48 μM for *C. butyricum* (*p* = 0.04); 123.2 ± 36.18 μM for *D. hominis* (*p =* 0.02) vs. 207.4 ± 23.11 μM for vehicle-treated mice; Fig. 3c). Finally, to further confirm succinate consumption ability, we tested these strains in a YCFA medium supplemented with 1% succinate, and all three showed similar succinate consumption rates in vitro (Supplementary Fig. S1: Additional file 17).

Based on these analyses, *D. hominis* and *O. laneus* emerged as the best candidates. We selected *O. laneus* for further study for three reasons. First, based on our published data in human cohorts, the *Odoribacteraceae* family is one of two succinate-consuming bacterial families associated with circulating succinate [12]; second, *D. hominis* was not detected in the human samples from our cohort; third, *O. laneus* exhibited the strongest succinate-consuming phenotype in FBA analysis in gut conditions and also appeared to have an impact on body weight (Fig. 3a). We next performed a metataxonomic analysis before and after probiotic intervention to screen for differential microbial abundance. We confirmed that *O. laneus* was enriched in the cecum of orally treated mice (Supplementary Fig. S2A). No relevant differences were found at the phylum level, but in an analysis of the top 10 more abundant taxa, we found a significant increase in *Prevotellaceae* families (9.6 ± 2.23% for vehicle versus 11.66 ± 4.05% for *O. laneus*-treated) and *Rikenellaceae* (10.51 ± 3.09% for vehicle versus 12.26 ± 3.27% for *O. laneus*-treated). The same analysis at the genus and species levels revealed significant changes in members of these families but also in some members of *Muribaculaceae* (20.97 ± 3.55% for vehicle versus 24.08 ± 5.51% for *O. laneus*-treated, at the genus level; Fig. 3d and Additional files 1, 2, 3, 4, 5, 6, 7, and 8). Notably, the expression of pro-inflammatory markers in various tissues of *db/db* mice administered orally with *O. laneus* was significantly lower than in vehicle-treated mice, and this was particularly evident in WAT (Fig. 3e). As a control for these analyses, we repeated the experiments with heat-inactivated *O. laneus*, which demonstrated that bacterial metabolism was needed for the evident reduction in circulating succinate and proinflammatory markers (Supplementary Fig. S2 B–E: Additional file 17)*.*

**Fig. 4:**
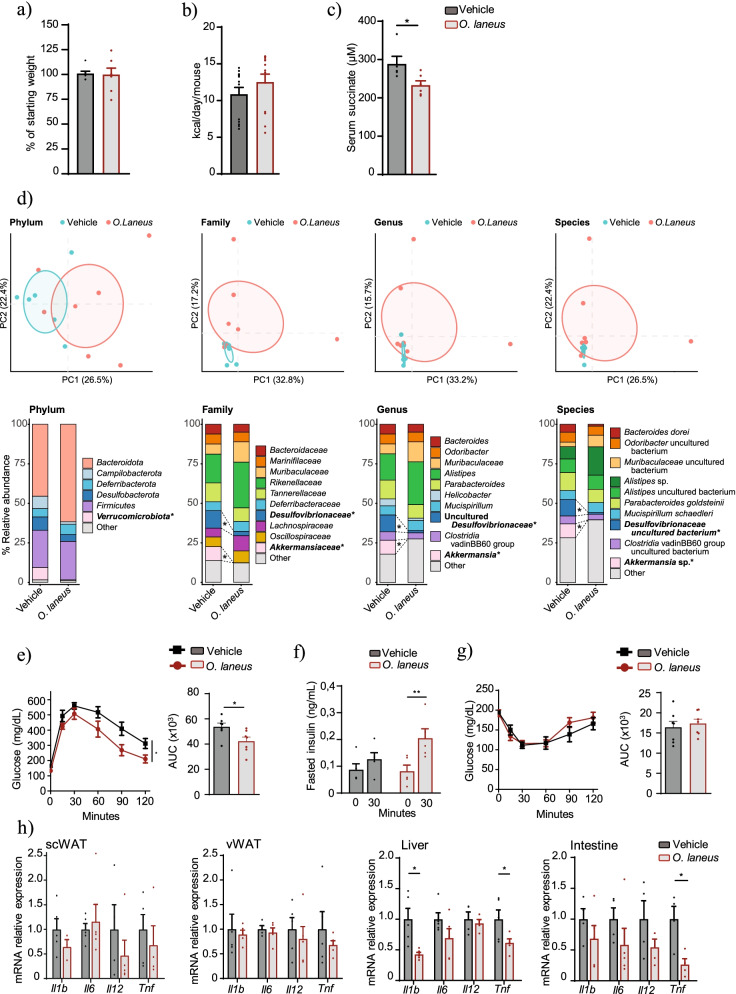
Probiotic intervention with *Odoribacter laneus* ameliorates glucose tolerance and inflammation in mice with diet-induced obesity. Weight evolution (**a**) and food consumption (**b**) during probiotic treatment (*n* = 7). Fasted serum succinate levels (*n* = 5–6) (**c**). Principal component analysis and average phylum, family, genera, and species abundance of the 10 more abundant taxa in the cecum of DIO mice treated with vehicle or *O. laneus* (**d**) (*n* = 6–8). Glucose tolerance test (**e**) (*n* = 7). Insulin secretion during glucose tolerance test (**f**) (*n* = 5). Insulin tolerance test (**g**) (*n* = 7). mRNA expression levels of inflammatory genes in the scWAT, vWAT, liver, and intestine (**h**) (*n* = 5). Data are presented as mean + s.e.m. *p < 0.05; **p < 0.01 (unpaired *t*-test and two-way ANOVA)

We complemented this analysis using the DIO mouse model, which better resembles the pathophysiology of human obesity. No differences were observed for body weight evolution or food consumption between DIO mice administered or not with *O. laneus* (Fig. 4a, b); however, reflecting the results from *db/db* mice (Fig. 3c), *O. laneus* administration led to a significant decrease in circulating succinate in DIO mice (233.27 ± 11.11 μM for *O. laneus* vs. 288.7 ± 19.88 μM (*p* = 0.03) for vehicle; Fig. 4c). Changes in cecal microbiota composition (top 10 taxa) after probiotic treatment were different in the DIO model than in the *db/db* model, including a decreased proportion of the *Verrucomicrobiota* phylum (7.9 ± 7.28% for vehicle versus 0.55 ± 1.43% for *O. laneus*-treated). At the family level, we also found a significant decrease in *Desulfovibrionaceae* (11.40 ± 7.88% for vehicle versus 2.45 ± 3.80% for *O. laneus*-treated) and *Akkermansiaceae* (8.54 ± 6.59% for vehicle versus 0 ± 0.01% for *O. laneus*-treated; Fig. 4d and Additional files 9, 10, 11, 12, 13, 14, 15, and 16). Notably, the decrease in succinate induced by orally administered *O. laneus* was accompanied by a significant improvement in glucose tolerance (Fig. 4e) and was associated with a greater stimulation of insulin secretion (measured 30 min after an intraperitoneal glucose bolus) (Fig. 4f). No differences were detected in insulin sensitivity between DIO mice administered or not with *O. laneus*, as revealed by an insulin tolerance test (Fig. 4g). Finally, mRNA analysis showed an overall decrease in the expression of various proinflammatory markers in *O. laneus-*administered DIO mice, which was particularly pronounced for intestinal and hepatic *Tnf* and hepatic *Il1b* (Fig. 4h). Overall, these data indicate that *O. laneus* is a succinate-consuming bacterium with beneficial anti-inflammatory and metabolic properties in the setting of obesity in mice.

### The beneficial metabolic effects of *O. laneus* are partly mediated by SUCNR1

**Fig. 5:**
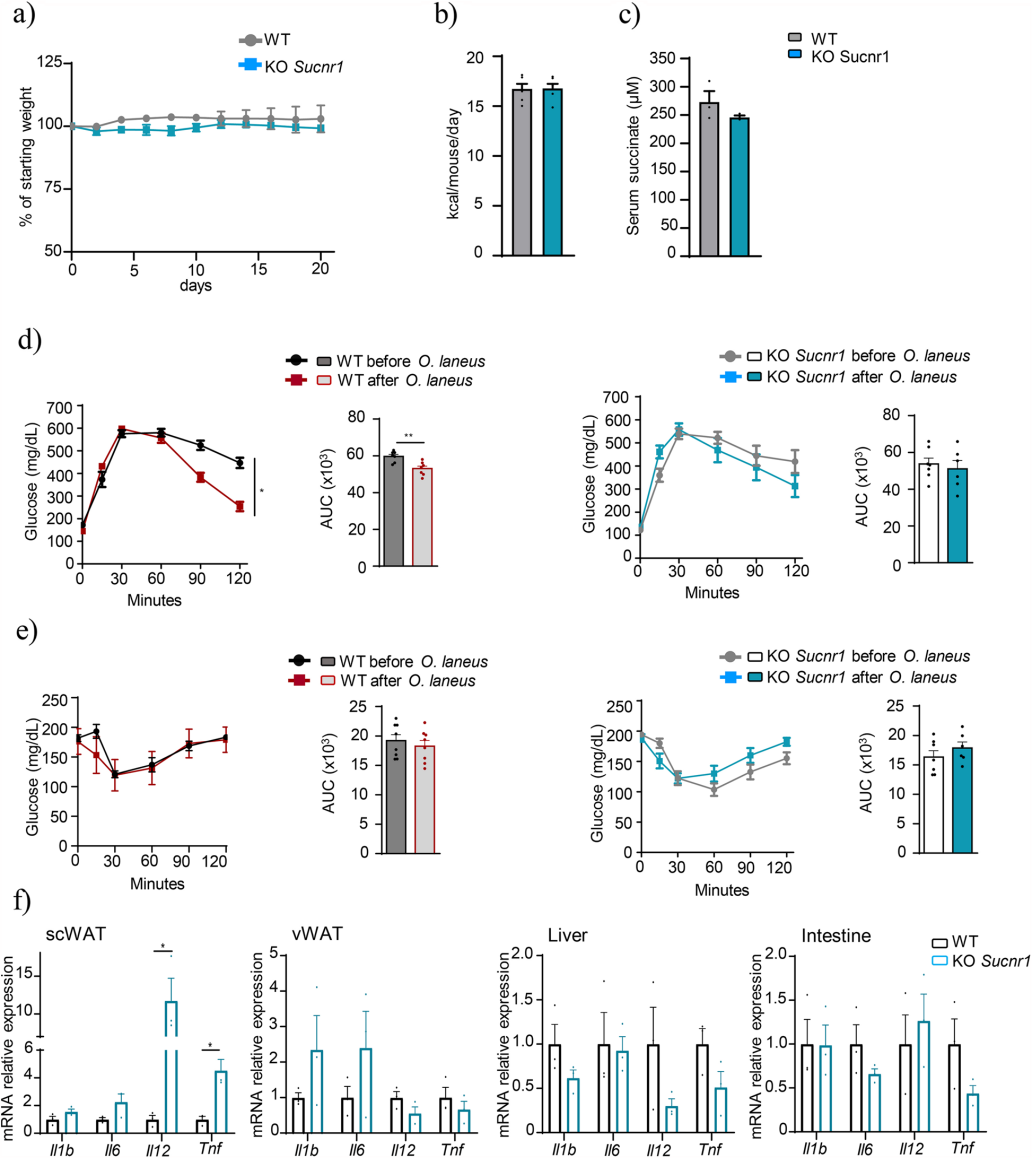
Probiotic intervention with *Odoribacter laneus* has no effect in *Sucnr1* knock-out mice. Weight evolution (**a**), food intake (**b**), and fasted serum succinate levels (**c**) of C57BL/6 wild type (WT) and *Sucnr1* knock-out (KO) mice treated with *O. laneus*. Glucose (**d**) and insulin (**e**) tolerance tests of WT and *Sucnr1* KO mice before and after probiotic treatment. mRNA expression levels of inflammatory genes in the scWAT, vWAT, liver, and intestine (**f**) (*n* = 7–8). Data are presented as mean + s.e.m. *p < 0.05; (unpaired *t*-test and two-way ANOVA)

To test whether the beneficial metabolic effects of *O. laneus* are dependent on succinate as an extracellular signaling molecule, we repeated the probiotic intervention in wild-type and *Sucnr1* knock-out mice after 8 weeks of HFD to induce obesity. No changes in body weight evolution, food intake, or serum succinate levels were observed between the two groups of mice during the probiotic intervention (Fig. 5a–c). By contrast, the improvement in glucose tolerance induced by the probiotic intervention in wild-type obese mice (Figs. 4e and 5d) was not observed in *Sucnr1* knock-out mice (Fig. 5d, right panel). This lack of response was not due to differences in systemic succinate levels measured at the end of the treatment regimen (272.9 ± 19.46 μM in wild-type vs. 245.77 ± 3.32 μM in *Sucnr1* knock-out; *p* = 0.24, Fig. 5c). No differences were found for insulin resistance between the two groups of mice as measured by an insulin tolerance test (Fig. 5e).

Analysis of the anti-inflammatory effects of *O. laneus* treatment revealed several differences between wild-type and *Sucnr1* knock-out mice (Fig. 5f). We found higher expression of some inflammatory markers in scWAT and vWAT from *Sucnr1* knock-out mice than from wild-type mice (significant for *Il12* and *Tnf* in scWAT), which is in good agreement with the anti-inflammatory role described for SUCNR1 in adipose-tissue resident macrophages in subcutaneous fat depots [15]. These results demonstrate that the beneficial metabolic and anti-inflammatory effects of *O. laneus* in obese mice are associated with its ability to consume succinate and are partly dependent on SUCNR1 signaling.

### Plasma succinate inversely correlates with fecal succinate and is a determinant of insulin sensitivity in patients with severe obesity

**Fig. 6:**
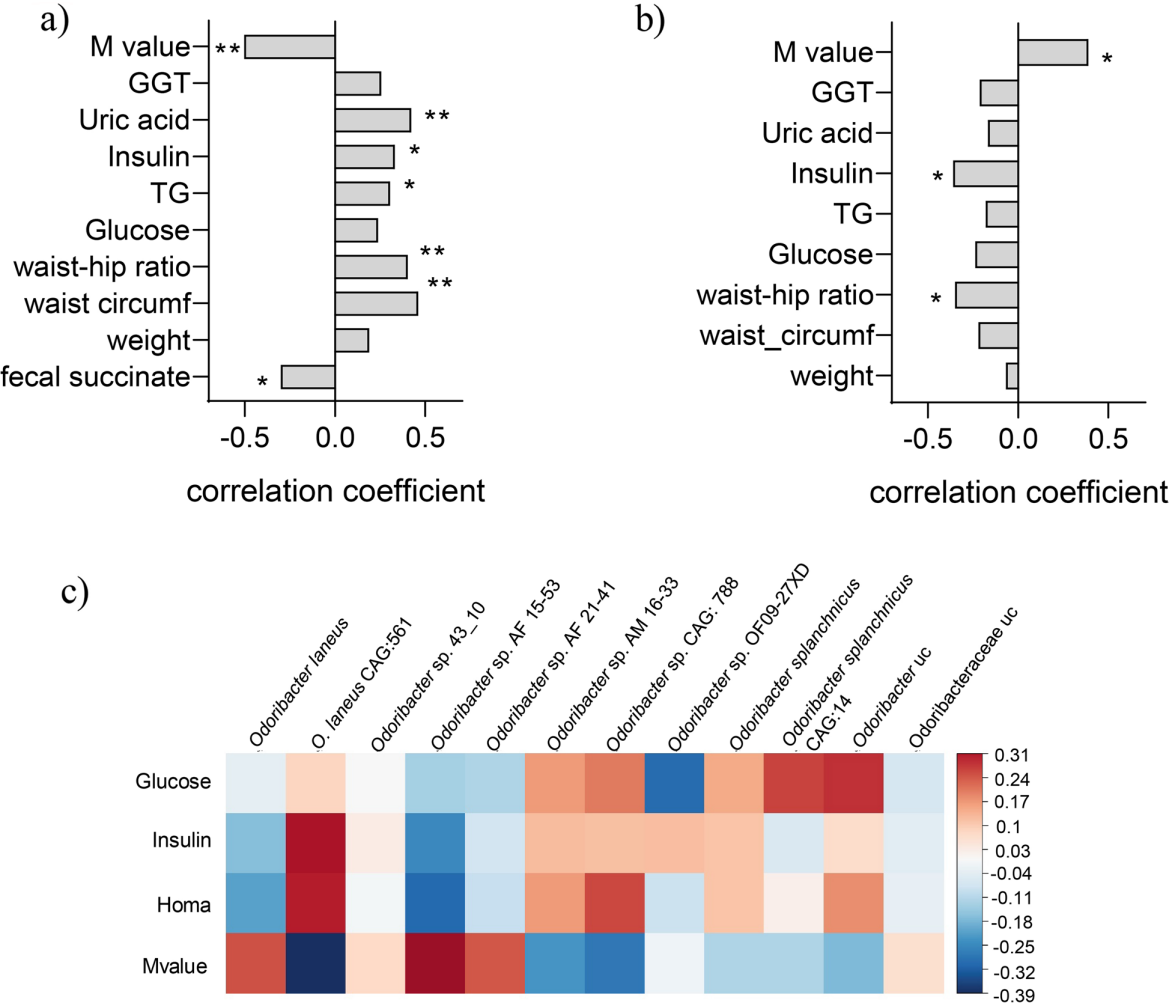
Plasma and fecal succinate levels and presence of *Odoribacteraceae* in a cohort of morbidly obese patients in association with anthropometric and metabolic parameters. Kendall’s tau_b correlation coefficients between plasma (**a**) or fecal (**b**) succinate and different metabolic and anthropometric parameters. Correlation heatmap of host metabolic parameters and clr-transformed *Odoribacteraceae* species (**c**) (*n* = 25). *p < 0.05; **p < 0.01

To validate the in vivo mouse data, we measured circulating plasma and fecal succinate in 25 patients with severe obesity (Table 1). By applying a rank-based approach based on Kendall’s Tau, we found a negative correlation between fecal and circulating succinate levels (*r* = −0.301, *p* = 0.045, Fig. 6a), which is consistent with the mouse data (see Fig. 1d–g and Supplementary Fig. S1: Additional file 17) and suggests succinate flooding into circulation in an obesity setting.

We found that higher circulating succinate levels in patients with severe obesity were associated with a worse metabolic profile, as previously described in other cohorts [12, 49]. Specifically, we observed significant positive correlations between circulating succinate levels and waist circumference (*r* = 0.461, *p* = 0.001), waist-to-hip ratio (*r* = 0.403, *p* = 0.005), triglycerides (*r* = 0.306, *p* = 0.033), insulin (*r* = 0.331, *p* = 0.021), and uric acid (*r* = 0.423, *p* = 0.003). In addition, plasma succinate correlated negatively with the *M*-value (*r* = −0.503, *p* = 0.001), a marker for insulin sensitivity measured by the euglycemic-hyperinsulinemic clamp (Fig. 6a). Fecal succinate was negatively associated with waist-to-hip ratio (*r* = −0.349, *p* = 0.021) and insulin (*r* = −0.360, *p* = 0.016) and positively associated with the M-value (*r* = 0.389, *p* = 0.016) (Fig. 6b). We then developed a linear regression model to evaluate the independent factors associated with insulin sensitivity, finding that plasma (but not fecal) succinate and HbA1c were the main independent factors associated with insulin sensitivity (Table 2).

**Table 2:** Linear regression model for the prediction of insulin sensitivity (*M*-value)

*M*-value (*R* = 0.747; *R*^2^ = 0.512)					
	*B* (unstandardized)	SE	95% CI	Beta (standardized)	*p*
Constant	29.984	8.870	15.606 to 44.363	-	<0.001
Plasma succinate	−0.071	0.028	−0.129 to −0.012	−0.444	0.022
HbA1c	−3.377	1.441	−6.394 to −0.360	−0.415	0.030

Finally, we performed a metagenomic analysis to test for the presence of the *Odoribacteraceae* family in the same human cohort. Although we failed to find any significant correlation between succinate and the *Odoribacteraceae* family or specific *Odoribacter* sp., *O. laneus* behaved differently from other members of the family, showing an inverse linear trend with glucose, insulin, and HOMA and a positive trend with *M*-value (Fig. 6c).

**Fig. 7:**
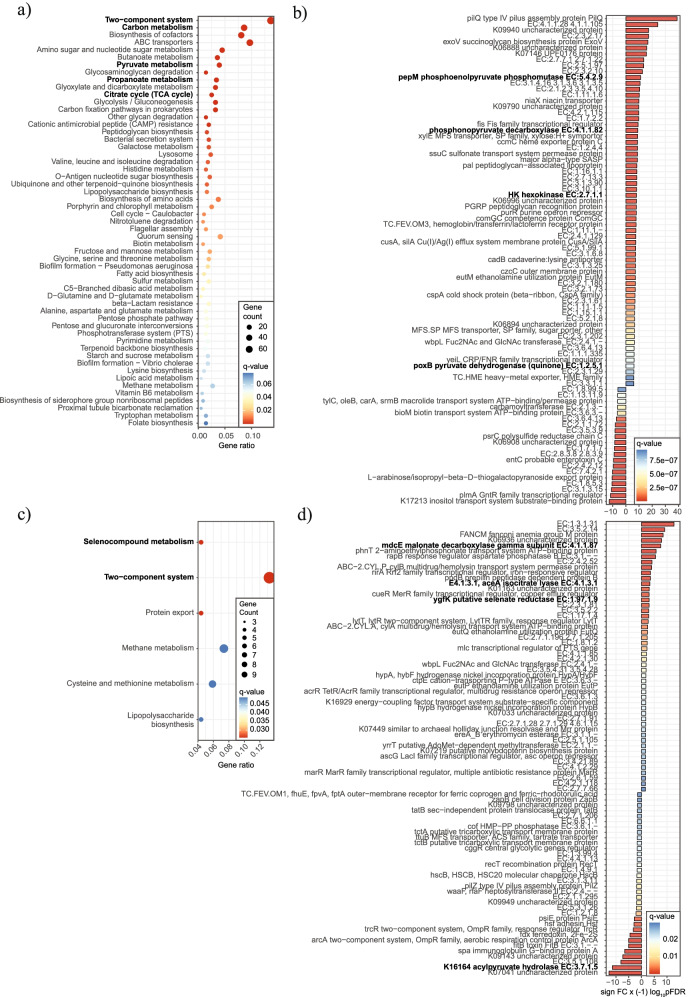
Plasma and fecal succinate linked to metagenomic functions. Dotplot (**a**) and Manhattan-like plot (**b**) showing the significantly expressed KEGG metagenome functions associated with plasma succinate. Dotplot (**c**) and Manhattan-like plot (**d**) showing the significantly expressed KEGG metagenome functions associated with fecal succinate

We next conducted functional analyses in the cohort of patients with obesity. Metagenome functional analyses based on KEGG pathways controlling for age, BMI, and sex and using over-representation analysis (ORA) revealed an association between fecal succinate and several significantly expressed KEGG metagenome functions related to succinate metabolism, including the two-component system and carbon, pyruvate, and propanoate metabolism (shown in bold in Fig. 7a). Significant changes were found in microbial genes linked to such functions including phosphonopyruvate decarboxylase, phosphonolpyruvate phosphomutase, and hexokinase and pyruvate dehydrogenase (Fig. 7b). We also performed a more specific metagenomic analysis on Krebs cycle genes, which revealed a clear upregulation of several key genes related to succinate production, specifically dihydrolipoyllysine-residue succinyltransferase (DLST, also known as α-ketoglutarate dehydrogenase component), succinyl-coA synthetase, and the DLD homodimer, which functions as the E3 component of several dehydrogenase complexes including α-ketoglutarate dehydrogenase (Supplementary Fig. S3: Additional file 17).

Finally, we also searched for specific functional metagenomic signatures in association with plasma succinate. In this analysis, and mirroring the results observed for fecal succinate (Fig. 7a), the most significant change was detected in the two-component system, and selenocompound metabolism also appeared among the most significant altered functions (Fig. 7c). At the gene level, plasma succinate was significantly linked to an upregulation of isocitrate lyase, malonate decarboxylase, selenate reductase, and acylpyruvate hydrolase, among others (Fig. 7d).

Overall, our clinical data support the close association between gut microbiota functionality, intestinal and systemic succinate, and metabolic profile.

## Discussion

Circulating levels of succinate are chronically elevated in obesity and type 2 diabetes [12, 17], where it is accepted to play a relevant pathological role. The source of the hypersuccinemia remains, however, unclear. In the context of the gut microbiota, succinate has been traditionally considered solely as a cross-feeding intermediate, and its functional consequences have been understudied [50]. To the best of our knowledge, we show for the first time the specific contribution of intestinal microbiota to circulating succinate levels, particularly in the obesity context. Paradoxically, whereas obesity-related changes in gut microbiota have been linked to a specific microbiota signature characterized by a high proportion of succinate producers [12], our data show lower levels of fecal succinate in obese mice than in non-obese mice and a negative correlation between fecal and plasma succinate in patients with obesity. It is widely accepted that a certain degree of gut-produced metabolite leakiness into circulation occurs in different contexts [51, 52], which may reach significant peripheral concentrations and directly impact host metabolism [53]. Similar to what has been recently described for SCFAs and bile acids in patients with type 2 diabetes, where an inverse correlation between fecal and systemic levels has been observed [54], our data support the notion of a leaky gut-enhanced flooding of microbiota-derived succinate into circulation. Our observation of an increase in circulating succinate after succinate intracolonic administration also supports this concept.

As hypersuccinemia might be a driving force for inflammation, we sought to reduce the systemic levels of succinate using a microbiota-targeted therapy based on succinate consumers to ameliorate obesity-related features. From our initial list of 22 potential non-pathogenic succinate-consuming bacterial strains, only three were confirmed by FBA [24, 25]. Intriguingly, no additional bacteria exhibited a consumer phenotype, even when succinate was the sole non-limiting nutrient. We could not determine why the succinate-consuming phenotype was not observed for these bacteria, although one reason might be that our study did not include simulations of the metabolic interactions with other bacteria of the gut. However, when we tested 5 potential succinate-consuming strains in vivo, we found that *C. butyricum*, *O. laneus*, and *D. hominis* could significantly reduce circulating succinate in a model of obesity. Characterization of the probiotic potential of *O. laneus* (a bacterium initially isolated from a human healthy Japanese donor [55]) in two different murine models of obesity (*db/db* and DIO) revealed that the reduction in circulating succinate induced by this single strain is concomitant with an improvement of host inflammatory and metabolic profiles. Experiments using heat-inactivated *O. laneus* established that these effects depended on the metabolic activity of the bacterium. While classical species from the genus *Bifidobacterium* and *Lactobacillus* and other more recently described species (e.g., from the genus *Akkermansia* [56]) are recognized as probiotics able to improve glucose metabolism by yet-to-be-clarified molecular mechanisms, this is the first time that a member of the *Odoribacteraceae* family has emerged as a promising probiotic in vivo. Although *Odoribacter* sp. has been classically linked to animal-based diets [57, 58], our encouraging results align with recent findings showing a higher abundance of *Odoribacter* sp. in people on diets rich in folate and B-class vitamins, a group of vitamins that promote the growth of anti-inflammatory bacterial species [59], and with our recent study showing an increase in *Odoribacteraceae* abundance after a dietary intervention [12]. Indeed, anti-inflammatory actions have been identified for *Odoribacter* sp., such as those described for *O. splanchnicus* in gut epithelium cells in vitro [60], and a recent clinical study describing a negative correlation between *Odoribacter* sp. abundance in gut microbiota and insulin resistance in Japanese subjects [61].

Our metataxonomic analysis of gut microbiota after *O. laneus* probiotic intervention in mice revealed changes in microbial abundance between the *db/db* and DIO models, which is in accord with the different gut microbiome signatures described for genetic and diet-driven obesity [62]. The different microbiomes defined for humans and mice [63] make it, however, difficult to extrapolate these results to a human setting. Unexpectedly, we observed, specifically in the DIO model, a reduction in the abundance of *Akkermasia* sp., which has been linked to positive metabolic effects. Nonetheless, it should be noted that *A. muciniphila*, which is the species most widely associated with metabolic health [56, 64], was not detected in our samples, and some authors have described detrimental effects of other members of this genus in some pathological contexts [65].

Our results contrast with recent studies showing that some probiotic interventions with bacterial succinate producers might be effective for improving obesity-related metabolic disturbances. For instance, it has been described that the established succinate producer *Prevotella copri* can increase cecal but not portal succinate, which is used as an intestinal gluconeogenic substrate under basal conditions [9]. It also appears that *P. copri* improves glucose tolerance [10], with the caveat that the same authors indicated that not all the beneficial effects observed for *Prevotella* were attributable to succinate metabolism [10]. In an obesity context, colonization with other succinate producers such as *Parabacteroides distasonis* has also shown health benefits in mice, although the authors also identified secondary bile acids as a potential mechanism involved in the metabolic beneficial effects of this strain [66]. While this might seem contradictory, these findings fit well with our results if a distinction is made between normal physiological and aberrantly high succinate levels. Indeed, in our perspective, succinate pleiotropy is well explained by the amount of succinate available: low, physiological levels of succinate have beneficial and homeostatic effects in a healthy setting, as for example improving glycemic control through activation of intestinal gluconeogenesis [9], whereas an excess of succinate is generated in different pathological scenarios where it plays a damaging pro-inflammatory role, not only in the context of obesity-related disorders and its complications [4, 67–69] but also in other diseases such as IBD [8]. Similarly, this dichotomy has also been reported for some SCFAs such as propionic acid, which although defined as an anti-inflammatory factor, is significantly increased in patients with type 2 diabetes and is linked to gut barrier disruption [54].

Our data in *Sucnr1* knock-out mice indicate that the observed beneficial metabolic effect of *O. laneus* depends, at least partly, on succinate depletion and is linked to SUCNR1 activation. Moreover, while seemingly contradictory, the increase in pro-inflammatory marker gene expression in *Sucnr1* knock-out mice agrees with our previous findings showing that SUCNR1 deficiency in macrophages induces a pro-inflammatory phenotype [15] as a consequence of its function in anti-inflammatory macrophages [15, 70]. Overall, our data fit with our initial hypothesis, which presupposed that a reduction in the aberrantly high levels of succinate would have beneficial effects, at least in terms of inflammation.

The extent to which other previously reported microbial metabolites, including SCFAs, contribute to the improvement in the metabolic and inflammatory profile remains to be elucidated. Also, it is important to highlight that there is clear evidence of an immunomodulatory role of the succinate-SUCNR1 axis in the intestine [11, 71, 72], which might also contribute to the observed effects. Accordingly, the relative contribution of local (intestinal) and leaked succinate to the overall metabolic and anti-inflammatory effects warrants further research. Our data do not rule out either the possibility of additional positive effects on immunoregulation driven by other effectors, such as those reported for *O. splanchnicus* [60], as we did not specifically address this point.

In accordance with the in vivo mouse data, we found that circulating and fecal succinate were inversely correlated in patients with severe obesity, suggesting that intestinal succinate might be released into circulation. Indeed, intrarectal succinate instillation induces an increase of circulating succinate. Succinate was introduced intrarectally rather than orally because colonic absorption provides a more direct route for succinate to reach the systemic circulation, mirroring the location of microbiota-derived succinate. Thus, our data supports the notion that similar to SCFA, the succinate produced by the gut microbiota could cross the intestinal barrier and reach the systemic circulation. Of interest was also our finding that plasma succinate was independently associated with the *M*-value (the lower the value of circulating succinate, the higher the insulin sensitivity). By contrast, fecal succinate levels were positively correlated with insulin sensitivity, with plasma succinate being a main determinant of the *M*-value. Thus, we establish for the first time a link between systemic and fecal succinate and insulin sensitivity. Along this line, increased succinate levels in skeletal muscle have been related to an improvement in insulin sensitivity [73].

Although no significant correlation was found between *Odoribacteraceae* or *Odoribacter* sp. and metabolic parameters in human samples, *O. laneus* behaved differently from many other members of the *Odoribacteraceae* family, pointing to a strain-specific effect, as previously described for other probiotics [74]. Still, it is important to consider that the identification of novel probiotics has to reconcile the limitation of a naturally different microbiome between humans and rodents [63], and while obesity and type 2 diabetes are twin disorders with an overlapping pathophysiology, global inflammation, different diets, and dissimilar degrees of metabolic impairment may also account for the distinctive microbiome profiles.

Finally, our study on the metagenomic functions associated with plasma and fecal succinate in a human cohort of obese patients revealed clear associations between succinate and several bacterial functions. Both fecal and plasma succinate were linked to significant changes in the two-component system, which is key in the bacterial response to C_4_-dicarboxylates. Interestingly, plasma succinate was associated with several KEGG functions directly related to succinate metabolism, such as malonate decarboxylase (EC:4.1.1.87), which inhibits succinate dehydrogenase, and isocitrate lyase (EC:4.1.3.1), and others without a direct link that might be the consequence of indirect interactions. Notably, several genes involved in selenate metabolism such as selenate reductase (EC:1.97.1.9) were found to be significantly linked to succinate and a recent publication has described selenium as a mediator of metabolic reprograming that facilitates anti-inflammation and pro-resolution [75]. Our analysis also revealed an upregulation of genes related to the phosphoenolpyruvate (PEP) node, being the most relevant phosphonopyruvate decarboxylase, which carboxylates PEP to oxalacetate and is the primary fermentative route for succinate [76] Remarkably, our study on Krebs cycle-related genes in association with fecal succinate revealed several genes significantly upregulated, with DLST (EC:2.3.1.61) and succinyl-coA synthetase (EC:6.2.1.5) being the most significant. DLST is the E2 transferase of the α-ketoglutarate dehydrogenase complex, which converts α-ketoglutarate to succinyl-CoA in the Krebs cycle, whereas succinylcoA synthetase converts succinyl-coA to succinate. Both enzymes are upstream of succinate and boost succinate production.

In conclusion, we show that microbiota-derived succinate is not merely a cross-feeding metabolite. We demonstrate that when the gut microbiota is altered with increased intestinal permeability, succinate can cross the epithelium and reach systemic circulation, negatively influencing host metabolism and inflammatory status. An intervention with the succinate-consuming bacteria *O. laneus* can effectively counteract such effects, thus emerging as a potential next-generation probiotic candidate for systemic succinate reduction.

## Supplementary Information

**Additional file 1.**  Abundance  of microbial taxa in *db/db* mice at phylum level.

**Additional file 2.** Abundance of microbial taxa in *db/db* mice at family level

**Additional file 3.**  Abundance of microbial taxa in *db/db* mice at genus level.

**Additional file 4.** Abundance of microbial taxa in *db/db* mice at species level.

**Additional file 5.** ANCOM analysis of compositions of microbiomes with bias correction (ANCOM-BC 1.2.2) R package in *db/db* mice at phylum level.

**Additional file 6.** ANCOM analysis of compositions of microbiomes with bias correction (ANCOM-BC 1.2.2) R package in *db/db* mice at family level.

**Additional file 7.** ANCOM analysis of compositions of microbiomes with bias correction (ANCOM-BC 1.2.2) R package in *db/db* mice at genus level.

**Additional file 8.** ANCOM analysis of compositions of microbiomes with bias correction (ANCOM-BC 1.2.2) R package in *db/db* mice at species level.

**Additional file 9.** Abundance of microbial taxa in DIO mice at phylum level.

**Additional file 10.** Abundance of microbial taxa in DIO mice at family level.

**Additional file 11.** Abundance of microbial taxa in DIO mice at genus level.

**Additional file 12.** Abundance of microbial taxa in DIO mice at species level.

**Additional file 13.** ANCON analysis of compositions of microbiomes with bias correction (ANCOM-BC 1.2.2) R package in DIO mice at phylum level.

**Additional file 14.** ANCON analysis of compositions of microbiomes with bias correction (ANCOM-BC 1.2.2) R package in DIO mice at family level.

**Additional file 15.** ANCOM analysis of compositions of microbiomes with bias correction (ANCOM-BC 1.2.2) R package in DIO mice at genus level

**Additional file 16.** ANCOM analysis of compositions of microbiomes with bias correction (ANCOM-BC 1.2.2) R package in DIO mice at species level.

**Additional file 17.** Supplementary Figures and Tables.

## Data Availability

The raw sequencing data files supporting the results of this article are available in the European Nucleotide Archive under accession numbers PRJEB47736 and PRJEB47655.

## Abbreviations

BMI: Body mass index
CD: Chow diet
Clr: Centered log-ratio
DSM: Deutsche Sammlung von Mikroorganismen
EI: Electronic impact
ESI: Electrospray ionization
FBA: Flux balance analysis
GC: Gas chromatography
GF: Germ-free
HbA1c: Glycated hemoglobin
HFD: High-fat diet
JCM: Japan Collection of Microorganisms
KEGG: Kyoto Encyclopedia of Genes and Genomes
LC: Liquid chromatography
QqQ MS: Triple-quadruple mass spectrometry
SCFAs: Short-chain fatty acids
scWAT: Subcutaneous white adipose tissue
SUCNR1: Succinate receptor 1
vWAT: Visceral white adipose tissue
YCFA: Yeast casitone fatty acids
