# Supplementary material for: Orally administered Odoribacter laneus improves glucose control and inflammatory profile in obese mice by depleting circulating succinate
Source: Microbiome. 2022 Aug 25;10:135. doi: 10.1186/s40168-022-01306-y (PMC9404562; doi:10.1186/s40168-022-01306-y)
Supplement: Supplementary file 18 — Additional file 17. Supplementary Figures and Tables. [file 40168_2022_1306_MOESM17_ESM.pdf]

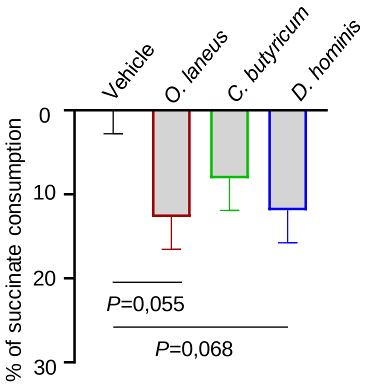

Supplementary Figure S1: *In vitro* succinate consumption rates. *O. laneus*, *D. hominis* and *C. butyricum* were tested in YCFA medium supplemented with 1% succinate Percentage of consumed succinate is shown (n=3). Data are presented as mean + s.e.m (unpaired t-test)

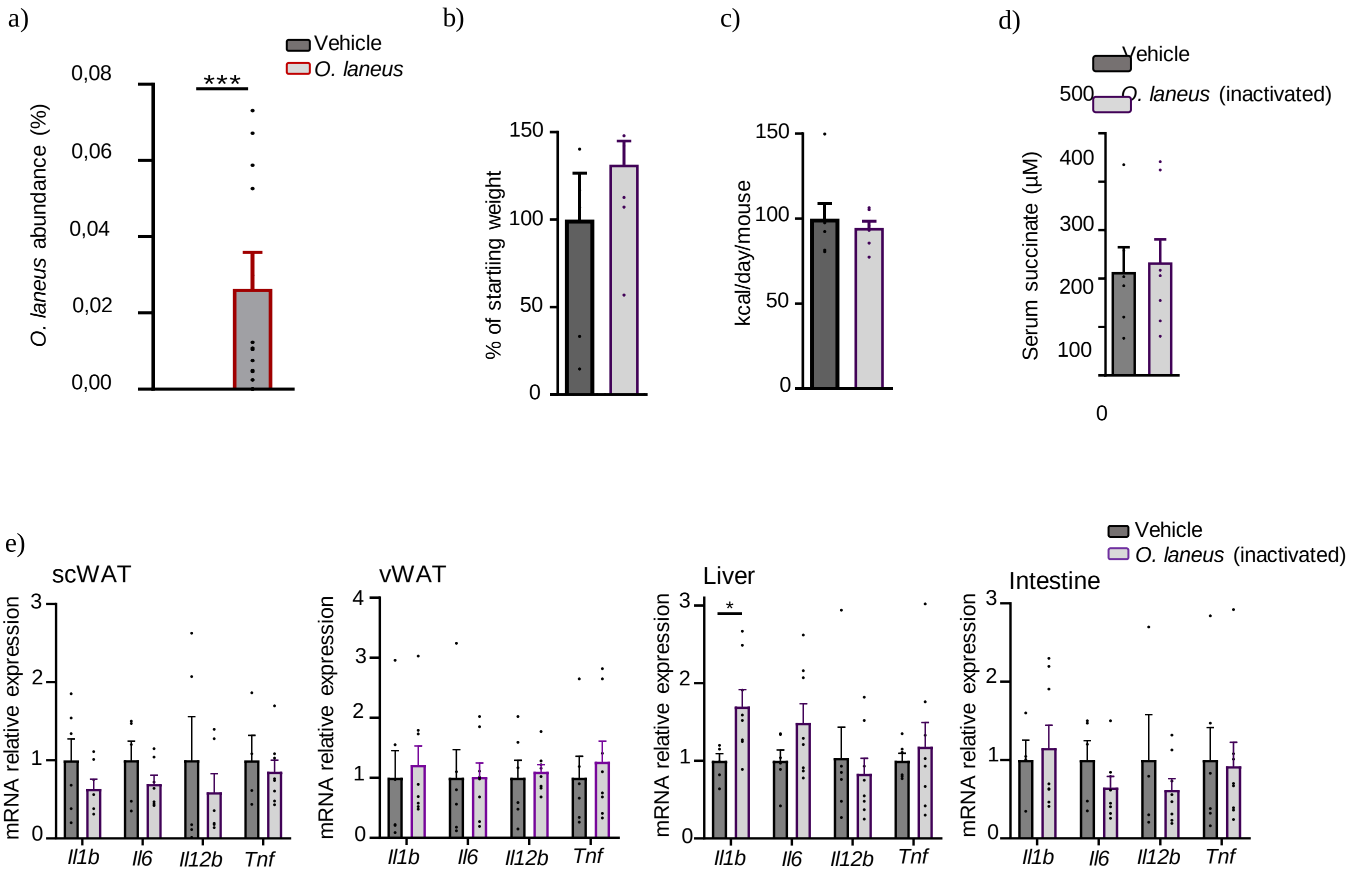

Supplementary Figure S2: Probiotic administration of inactivated *O. laneus* does not reduce circulating succinate or inflammation in *db/db* mice. Probiotic intervention with *Odoribacter laneus* increases the abundance of *O. laneus* in cecum of *db/db* mice treated with vehicle or with *O. laneus*. (n = 15). (a). Changes in body weight (b), food consumption (c), and fasted succinate serum levels (d) after administration of inactivated *O. laneus* (n = 6–8). Mean mRNA expression levels of inflammatory genes in scWAT, vWAT, liver and intestine (e). (n = 4–8). Data are presented as mean + s.e.m.; *p < 0.05 ***p<0.001 (unpaired t-test)

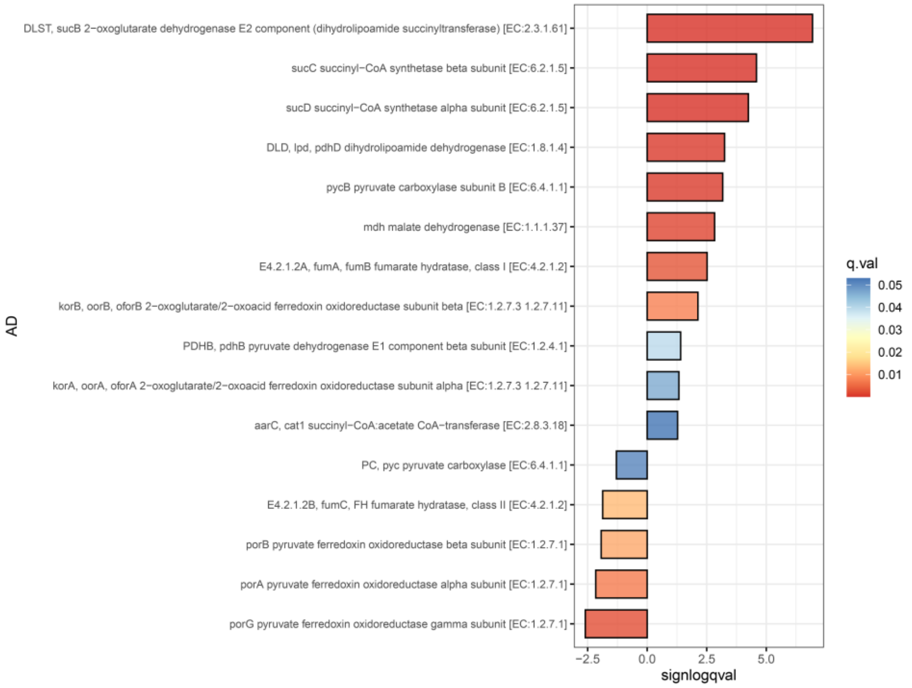

Supplementary Figure S3: Metagenomic study of TCA genes. Manhattan-like plot of significantly expressed KEGG metagenome functions of the TCA cycle associated with fecal succinate.

Detector	Taqman probes	Gene probes
B2m	Mm00437762_m1	Beta-2-microglobulin
Il1b	Mm00434228_m1	Interleukin 1 Beta
Il6	Mm00446190_m1	Interleukin 6
Il12b	Mm01288989_m1	Interleukin 12
Tnf	Mm00443258_m1	Tumor necrosis factor alpha

Supplementary Table S1. Mouse gene expression Taqman probes. Results were calculated using the comparative Ct method and expressed relative to the expression of the housekeeping gene *B2m*.

SPECIES	FAMILY	PHYLUM	GROWTH RATE obtained from FBA (h^{-1})	Succinate production (positive) or consumption (negative) rate (mmol/gDW/h)
Acidaminococcus provencensis	*Acidaminococcaceae*	*Firmicutes*	0.308	41.655
Bacillus firmus DS1	*Bacillaceae*	*Firmicutes*	0.571	49.898
Bacillus flexus	*Bacillaceae*	*Firmicutes*	0.427	49.786
Bacteroides thetaiotaomicron	*Bacteroidaceae*	*Bacteroidetes*	0.506	38.725
Caecibacter massiliensis	*Veillonaceae*	*Firmicutes*	Not simulated	Not simulated
Dialister invisus	*Veillonaceae*	*Firmicutes*	0.211	47.009
Dialister propionicifaciens	*Veillonaceae*	*Firmicutes*	Not simulated	Not simulated
Dialister succinatiphilus	*Veillonaceae*	*Firmicutes*	0.307	-1.742
Emergencia timonensis	*Clostridiales*	*Firmicutes*	0.392	49.256
Eubacterium hallii	*Lachnospiraceae*	*Firmicutes*	0.405	14.073
Massilibacillus massiliensis	*Veillonaceae*	*Firmicutes*	0.348	49.143
Megamonas hypermegale	*Selenomonadaceae*	*Firmicutes*	0.301	49.922
Odoribacter laneus	*Odoribacteraceae*	*Bacteroidetes*	0.335	-47.071
Phascolarctobacterium faecium	*Acidaminococcaceae*	*Firmicutes*	0.174	27.163
Phascolarctobacterium succinatutens YIT 12067	*Acidaminococcaceae*	*Firmicutes*	0.221	38.505
Romboutsia weinsteinii	*Peptostreptococcaceae*	*Firmicutes*	0.316	46.318
Ruminococcus bromii	*Oscillospiraceae*	*Firmicutes*	0.258	8.205
Selenomonas felix	*Selenomonadaceae*	*Firmicutes*	0.419	-12.125
Spirosoma linguale DSM 74	*Cytophagaceae*	*Bacteroidetes*	0.436	0
Succiniclasticum ruminis	*Acidaminococcaceae*	*Firmicutes*	0.265	43.818
Sutterella parvirubra	*Sutterellaceae*	*Proteobacteria*	0.249	49.931
Veillonella parvula	*Veillonaceae*	*Firmicutes*	0.417	49.974

Supplementary Table S2: Succinate-consuming strains identified with computational genome-based metabolic modelling technology. 22 potential succinate-consuming non-pathogenic strains were identified. Growth rates (expressed in h^{-1}) and succinate production (positive) or consumption (negative), all obtained from Flux Balance Analysis, are shown. The FBA were performed in human gut conditions, i.e. using the western diet nutrient conditions previously described [27]. *C. massiliensis* and *D. proponifaciens* could not be simulated because there is no genome assembly available
